# Technique for ectopic intrathymic parathyroid adenoma: the uniportal subxiphoid approach

**DOI:** 10.1093/jscr/rjab520

**Published:** 2021-12-11

**Authors:** Marc Hartert, Jan Tripsky, Martin Huertgen

**Affiliations:** Department of Thoracic Surgery, Katholisches Klinikum Koblenz-Montabaur, Koblenz, Germany; Department of Thoracic Surgery, Katholisches Klinikum Koblenz-Montabaur, Koblenz, Germany; Department of Thoracic Surgery, Katholisches Klinikum Koblenz-Montabaur, Koblenz, Germany

## Abstract

Parathyroid adenomas (PAs) are the main cause for primary hyperparathyroidism with almost a quarter of them being ectopic, most likely located in the superior mediastinum within the thymus. Besides the challenge of their prompt and correct diagnosis, utmost care should be taken during surgical resection as leaving behind parathyroid tissue may result in metastasis and recurrence of hyperparathyroidism. With tumor excision via median sternotomy or thoracotomy being the conventional approaches for a long period, video-assisted thoracoscopic surgery (VATS) is of gaining popularity. As the lateral thoracic approach lacks in clarity on the contralateral mediastinum, the newest evolution in VATS—the supxiphoid approach—closes the gap to the insufficient intraoperative visibility and hence optimizes postoperative outcome. We hereby present the practicality of the uniportal subxiphoid resection of an ectopic mediastinal PA.

## INTRODUCTION

Primary hyperparathyroidism (PHPT) is defined as unsuppressed secretion of parathyroid hormone leading to a dysregulated bone and mineral metabolism and ultimately causing hypercalcemia [1]. The main reasons for its development are parathyroid adenomas (PAs) with an estimated prevalence of 1–4/1.000 in the general population, predominant in postmenopausal women. During embryogenesis, superior and inferior parathyroid glands accompany the thyroid gland in their detachment and descent from its pharyngeal origin [2–4]. As any developmental migration might get faulty, the parathyroid glands may both descent excessively (*ectopic parathyroids*) or fail to descent at all (*undescended parathyroids*). Ectopic mediastinal PAs (MEPA), which account for ~1–2% of all PAs, are recognized as major cause for persistent PHPT with severe clinical manifestations [5]. With surgical MEPA-resection as the only therapeutic option, key points for successful surgical treatment are: (i) an adequate preoperative imaging modality to accurately localize the ectopic PA and (ii) the radical resection of all possible ectopic PA-including mediastinal tissue [6, 7]. With modern imaging techniques as essential prerequisite, the uniportal subxiphoid approach is an excellent surgical technique for a complete ectopic mediastinal parathyroidectomy.

## CASE

**Figure 1:**
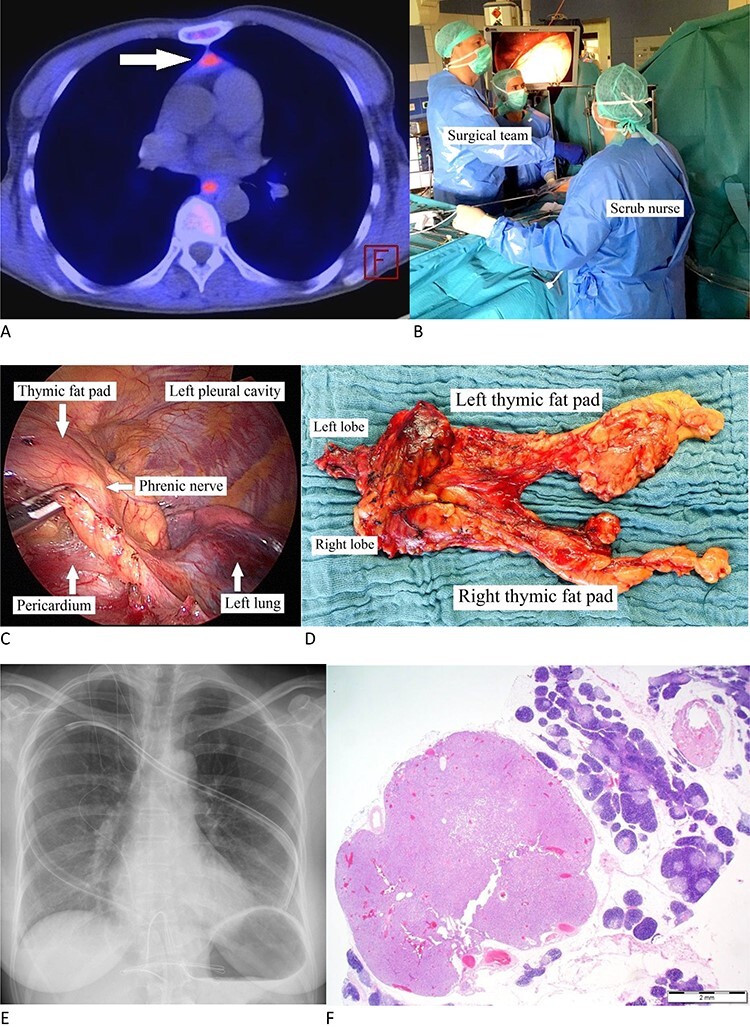
(**A**) Carbon-11-methionine PET-CT presenting a mediastinal ectopic PA (arrow). (**B**) Surgeon standing to the patient’s right with his assistant (camera operator) acting at his and the scrub nurse on the facing side. The video monitor is positioned towards anesthesia. (**C**) Thoracoscopic image: dissecting the pericardial fat pad alongside the phrenic nerve. (**D**) Resected specimen. (**E**) Postoperative X-ray. (**F**) PA is characterized by chief cells in acinar formations with a clear reduction in stromal fat content and focal clusters of oncocytic cells. Note the surrounding thymic tissue. (H&E stain, magnification ×1,25).

A 53-year-old female with a history of complete double-sided thyroidectomy with systematic bilateral cervical exploration on the basis of struma nodosa and PHPT three years earlier presented with persistent PHPT. Her initial symptoms (constipation, bladder irritability and irritable bowel syndrome) were slowly recurring under conservative therapy. Biochemical levels on admission are listed in Table 1. Even though Tc^99m^-sestamibi scanning was inconclusive, the suspicion of an MEPA was substantiated via carbon-11-methionine positron emission tomography-computed tomography (PET-CT; Fig. 1A). Multidisciplinary in-depth discussion involving radiologist, endocrinologist and thoracic surgeon resulted in recommendation for surgery in terms of the subxiphoid approach.

**Table 1:** Perioperative data

Characteristic	Value
**Preoperative findings** Age in years Gender Body mass index (in kg/m^2^) FEV_1_ in % Preoperative biochemical levels Serum calcium (in mmol/l) ^1^ Serum parathyroid hormone (in pg/ml) ^2^	53 Female 20.76 96.1 3.01 119
**Perioperative findings**	
Operative time (in min) Intraoperative blood loss (in ml)	159 50
Duration of chest tube drainage (in d) Duration of ICU-Stay (in d) Amount of pleural effusion (in ml) Postoperative stay (in d) Postoperative biochemical levels Serum calcium (in mmol/l) ^1^ Serum parathyroid hormone (in pg/ml) ^2^ NRS Postoperative Day 1 Postoperative Day 3 Postoperative Day 7 Follow-up (in month)	2 1 675 4 2.25 18.0 3 1 0 42
**Histopathological findings**	
Weight of resected specimen (in g) Maximum diameter of the PA (in mm) Extension of the specimen (length/width/height, in cm)	155.2 7:6:8 19.0:9.5:2.5

*Note:* FEV_1_ = forced expiratory volume; ^1^ standard value: 2.1–2.5 mmol/l; ^2^ standard value: 10–57 pg/ml; NRS = numerical rating scale, ranging from 0 (no pain) to 10 (worst pain imaginable).

Positioned supine and under general anesthesia, a double-lumen endotracheal tube was used for intermittent selective one-lung ventilation. A 5-cm vertical supxihoid incision was made above the xiphoid process. The subcutaneous tissue and the medial fractions of the *Musculus rectus* abdominis were dissected and the xiphoid process completely removed. Following blunt manual dissection of the retrosternal space, a sternal retractor was placed under the sternum, facilitating enhanced access by elevating the anterior mediastinum from below. Carbon dioxide insufflation was not performed as the elevation of the sternum allowed sufficient visibility. Following insertion of a flexible wound retractor the whole dissection was then being performed through the subxiphoid incision (Fig. 1B).

Under selective left lung ventilation, the mediastinal pleura was dissected along the sternal surface up to the level of the right internal thoracic vein. The pericardial fat as well as the right epiphrenic fat pad was dissected from the pericardium and diaphragm with the right phrenic nerve being the dorsal boundary of dissection (Fig. 1C). The dissection of the pericardial fat is preceded upwards under thoracoscopic control in an en-bloc fashion, avoiding any attempt to dissect the suspected MEPA-containing tissue separately. As the left mediastinal pleura was opened at a relatively early stage of the procedure, the dissected specimen could be moved out of vision (i.e. from the right into the left chest cavity) in order to facilitate the maneuver substantially. Besides carefully dissecting the prepericardial fat pad, skeletonization of superior vena cava, both innominate veins and the aorta is an objective. With the thyro-thymic ligaments as the sole attachments from the cranial side, the upper poles were divided close to the thyroid gland. Dissection of the specimen along the left phrenic nerve was performed similarly as on the right side. Finally, the specimen was placed in an endobag and removed through the subxiphoid incision (Fig. 1D). A single chest tube was inserted through the subxiphoid incision (Fig. 1E). The subxiphoid incision was closed in standard manner. The patient was extubated immediately after the operation.

Overall operative time was 159 min with negligible intraoperative blood loss (Table 1). The postoperative course was uneventful, particularly no injury of the phrenic or recurrent laryngeal nerves or brachiocephalic vein. The chest tube was removed within 2 days. Overall hospital stay was 4 days. Postoperative pain intensity was low. The resected specimen covered an overall extension of 12.0 × 7.5 × 1.5 cm containing a 7 × 6 × 8-mm sized histopathologically confirmed PA (Fig. 1F). Early on both biochemical cure and lack of symptoms were obvious with an unremarkable 42-month follow-up.

## DISCUSSION

When it comes to decide which surgical technique is best in resecting tumors in the anterior mediastinum, the subxiphoidal approach is the most fashionable and the *number one player* in specialized thoracic centers [8–11]. With thymic pathologies being the most common surgical indications for this technique, it is quite consequential to apply it for rather rare mediastinal pathologies like MEPAs.

As in surgical resection of thymic pathologies, the phrasing ‘the more complete the resection, the better the clinical outcome’ is also in the resection of MEPAs rather a command than an advice [7, 12]. Of course, basic prerequisite for any surgical success are excellent preoperative imaging studies, which definitely contributed to the high success rate of parathyroidectomy in the cure of PHPT [13, 14]. But the main focus of any parathyroidectomy is on radicality. In this sense, the most striking advantage of the subxiphoid VATS approach is its cinematographic view. The surgeons view keeps in center-line with the patient’s body—comparable with that of a median sternotomy—without losing sight of vulnerable structures like the phrenic nerves. Due to this exceptional visualization, the complete removal of all possible PA-bearing mediastinal fat tissue is facilitated and concomitant surgical damage is reduced.

Which surgical discipline will perform minimal-invasive thoracic procedures for endocrine reasons? In principal, those patients—with our patient being no exemption—are initially presented to a general surgeon. Not being reliant on *fishing-in-foreign-waters*, but specialized minimal invasive thoracic procedures like the subxiphoid approach for mediastinal pathologies has to stay in the hands of specialized thoracic surgeons, who are experienced in this technique [15]. Keeping that in mind, a multidisciplinary setting of endocrinologist, endocrine surgeon and thoracic surgeon is highly recommended as the subxiphoid approach is expanding the surgical possibilities to achieve complete MEPA-resection.
